# Effect of Higher Silicon Content and Heat Treatment on Structure Evolution and High-Temperature Behaviour of Fe-28Al-15Si-2Mo Alloy

**DOI:** 10.3390/ma14113031

**Published:** 2021-06-02

**Authors:** Martin Švec, Věra Vodičková, Pavel Hanus, Petra Pazourková Prokopčáková, Libor Čamek, Jaromír Moravec

**Affiliations:** 1 Department of Technology, Faculty of Mechanical Engineering, Technical University of Liberec, 461 17 Liberec, Czech Republic; martin.svec@tul.cz (M.Š.); jaromir.moravec@tul.cz (J.M.); 2 Department of Material Science, Faculty of Mechanical Engineering, Technical University of Liberec, 461 17 Liberec, Czech Republic; pavel.hanus@tul.cz (P.H.); petra.prokopcakova@tul.cz (P.P.P.); 3 Department of Foundry Engineering, Brno University of Technology, 601 90 Brno, Czech Republic; camek@fme.vutbr.cz

**Keywords:** Fe_3_Al-based iron aluminide for high-temperature applications, high-temperature yield stress, coefficient of thermal expansion

## Abstract

This paper describes the structure and properties of cast Fe_3_Al-based alloy doped with 15 at. % of silicon and 2 at. % of molybdenum. The higher content of silicon is useful for the enhancement of high-temperature mechanical properties or corrosion resistance of iron aluminides but deteriorates their workability due to increased brittleness. It was found that the presence of both alloying elements leads to an increase of values of the high-temperature yield stress in compression. The heat treatment (annealing at 800 °C for 100 h) used for the achievement of phase stability causes the grain coarsening, so the values of the high-temperature yield stress in compression are lower at 600 °C and 700 °C in comparison to values measured for the as-cast state. This stabilization annealing significantly improves the workability/machinability of alloy. Furthermore, the higher silicon content positively affects the values of the thermal expansion coefficient that was found to be lower in the temperature range up to 600 °C compared to alloys with lower content of silicon.

## 1. Introduction

The Fe_3_Al-based iron aluminides are considered a suitable material for high-temperature application [1,2,3]. They have many benefits, such as low weight and excellent corrosion resistance, as well as resistance to an aggressive environment. In addition, due to the low price of raw materials, low-cost production appears to be advantageous. However, the poor ductility and workability of cast binary alloys at room temperature were an obstacle to wider use. The ductility at room temperature of the ordered Fe_3_Al and Fe–Al phases varies with the degree of ordering, with a relative maximum ductility of about 8% at about 28 at. % Al [4]. However, most of the mechanical or physical properties of iron aluminides are sensitive, among other things, to the presence of alloying elements, and thus, can be improved by using them [4,5].

In the past, a lot of effort has been devoted to the research of alloying element influence on the problematic properties of iron aluminides, especially on the brittleness suppression and increasing ductility. For example, the positive effect of chromium on the ductility of iron aluminides at room temperature has been found [4]. The high-temperature tensile strength can be enhanced by using elements with high solubility in solid solution such as chromium, silicon, or titanium [5,6], or by elements such as zirconium, niobium, or carbon that participate in the formation of the secondary phase particles [7,8,9,10].

Despite problems caused by their brittleness, good results have been achieved within the processing of iron aluminides produced by classic casting. The use of both the additives such as Cr or TiB [11,12,13], and the special technological processes [14], have been applied to obtain material with good processing and mechanical properties.

Silicon seems to be an interesting additive for an improvement of the high-temperature behaviour in all groups of iron aluminides. Although the addition of silicon to the Fe–Al system can cause the formation of brittle ternary FeSi phases, other properties can be positively influenced, e.g., the oxidation resistance, wear resistance, or, generally, thermal stability [15,16,17]. If the silicon content is higher (more than 10 at. %) in iron aluminides prepared by classic casting, the material brittleness increases significantly. This fact causes machinability problems, which are an obstacle not only during the preparation of the samples for tests of mechanical properties but generally during the processing of products. This aspect can be compensated using powder metallurgy processes, especially by connection with mechanical alloying [18,19,20]. However, this technology still remains more expensive than classic casting.

The positive effect of silicon on the oxidation resistance of Fe–Al intermetallic alloys prepared by mechanical alloying and spark plasma sintering was also reported [21]. It has been shown that the oxidation rate of Fe–Si–Al alloys is three to four orders lower than that of Fe–Al or Fe–Si alloys due to the combined effect of different protective layers. Also, the use of silicon in coatings might be an interesting option to enhance the high-temperature oxidation resistance of surfaces of TiAl-based intermetallics by forming a protective silicon-rich scale [22].

Regarding high-temperature mechanical properties, the positive effect of Si addition on creep resistance Fe_3_Al-based iron aluminides has been already reported [4]. The effect of silicon on the formation of the strengthening Al_4_C_3_ phase in Fe–Al—type iron aluminides with carbon addition was investigated [23]. The presence of aluminum carbide in the structure proved essential for the creep resistance improvement of this group of iron aluminides [24].

In contrast with research on the additives influence on mechanical or high-temperature mechanical properties, there is limited information about the effect of additive elements, such as silicon on the coefficient of thermal expansion (CTE) of iron aluminides. In the past, the effect of different elements—among other things, silicon—on the CTE of different types of aluminum alloys (Al–Si, Al–Si–Mg) was investigated [25]. It has been shown that the CTE of this type of alloy decreases with increasing the atomic percent of added silicon. A similar trend regarding the effect of silicon on the thermal expansion coefficient was also found for different types of steel (austenitic, ferritic, or martensitic) as well as for Ni-base nonmagnetic alloys [26]. On several iron and nickel aluminides, the thermal expansion coefficients have been measured and compared with the CTE of 316 stainless steel alloys. It was found that CTE values of the chromium-doped Fe_3_Al-based alloy were very close to being 316 stainless steel in the whole range of measured temperatures [27].

On the Fe_3_Al iron aluminides specifically, the CTE measurements were carried in a temperature range of 460–1200 °C. The effect of different alloying elements (also silicon in lower amounts) on the coefficient of thermal expansion was evaluated concerning the phase composition and structure of the alloy [28].

In terms of high-temperature mechanical properties, the effect of additives (Mo and Ti) on the high-temperature strength in compression has been described recently for the Fe_3_Al-type iron aluminide with lower silicon content (5 at. %) [1]. The higher content of silicon (more than 10 at. %) can be advantageous regarding the possibility of the improvement of alloy properties due to the strengthening of the solid solution. On the other hand, it is necessary to take into account that the higher silicon content deteriorates the processing properties of this type of alloy manufactured in an inexpensive way, i.e., by the classic casting. Suitable heat treatment could thus affect the structure state towards improving ductility and/or machinability of the alloy.

The reason for the investigation of these types of alloys is an effort to substitute high-alloyed steels widespread in high-temperature applications. These steels are doped by a high amount of “critical raw elements” (chromium, nickel, etc.). Iron aluminides doped with silicon have the potential to achieve similar strength at higher temperatures (up to 600 °C) as these steels, saving the “critical raw elements”.

The article aims to discuss the common effect of the higher content of silicon and heat treatment on the structure evolution of the Fe-28Al-15Si-2Mo alloy, and concurrently describe their influence on the high-temperature behaviour of the alloy. It was found that both silicon and molybdenum addition support increasing the values of high-temperature yield stress significantly.

## 2. Materials and Methods

Vacuum induction melting and casting were used to prepare the alloys (a detailed description of the casting process was done in [1]). The nominal chemical composition of the investigated alloys is summarized in Table 1.

The microstructure was studied by means of the scanning electron microscope (SEM) Tescan Mira 3 (Tescan, Brno, Czech Republic) in the state after oxide polishing. All figures were taken by the detector of secondary electrons (SE). Local chemical composition was determined by Energy Dispersive X-ray Spectroscopy (EDX—Oxford Instruments, High Wycombe, UK) for indicative knowledge about the phase structure of alloys. The precise phase composition was verified by X-ray diffraction (XRD) using a X’Pert^3^ Powder diffractometer (PANalytical, Almelo, Netherlands) in Bragg–Brentano geometry (CuKα radiation, λ = 1.5418 Å, U = 40 kV, I = 30 mA). The volume fraction of precipitates was determined using NIS—Elements 5.0 software (Laboratory Imaging, Prague, Czech Republic). The grain sizes of alloys were measured by means of Electron Backscatter Diffraction (EBSD) by Oxford Instruments Symmetry detector (Oxford Instruments, High Wycombe, UK). The process parameters were set: high-voltage 20 kV, measured area 5 mm × 5 mm, step size 5 μm.

The samples for the study of microstructure were oxide-polished and etched by suspension OP-S in the final step. The structure of all samples was studied in an as-cast state and also after heat treatment at 800 ± 5 °C for 100 h. The temperature and time of this stabilization annealing were chosen concerning the expected maximum work temperature of this type of material. Annealing was carried out in the vacuum furnace. Cooling down was performed in the furnace with a cooling rate of 5 °C/min.

The high-temperature compression yield stress σ_0.2_ was measured using TESTOMETRIC FS100CT (Testometric, Rochdale, UK) at temperatures of 20, 600, 700, and 800 °C. The accuracy of the temperature chamber of the device was ± 1 °C, and the used initial strain rate was 1.5 × 10^−4^ s^−1^. Samples for high-temperature tests with dimensions of 5 mm × 5 mm × 8 mm were prepared partly by spark machining and partly by mechanical cutting using a ATM Brillant 220 saw.

The Quenching dilatometer DIL 805L (TA Instruments, New Castle, DE, USA) was used to measure the thermal expansion coefficient. The dimensions of the samples were 4 mm in diameter and 10 mm on high. All the samples were prepared from the material in the annealed state (800 °C/100 h) due to its better workability (machinability mainly). There was a temperature cycle used: induction heating to temperature 1100 °C with a heating rate of 1 °C/s in vacuum (10^−4^ Pa), 60 s stamina at temperature, and cooling to room temperature with a cooling rate of 1 °C/s in inert He atmosphere.

## 3. Results

### 3.1. Structure of Investigated Alloys

#### 3.1.1. Structure of Alloys in the As-Cast State

**Figure 1:**
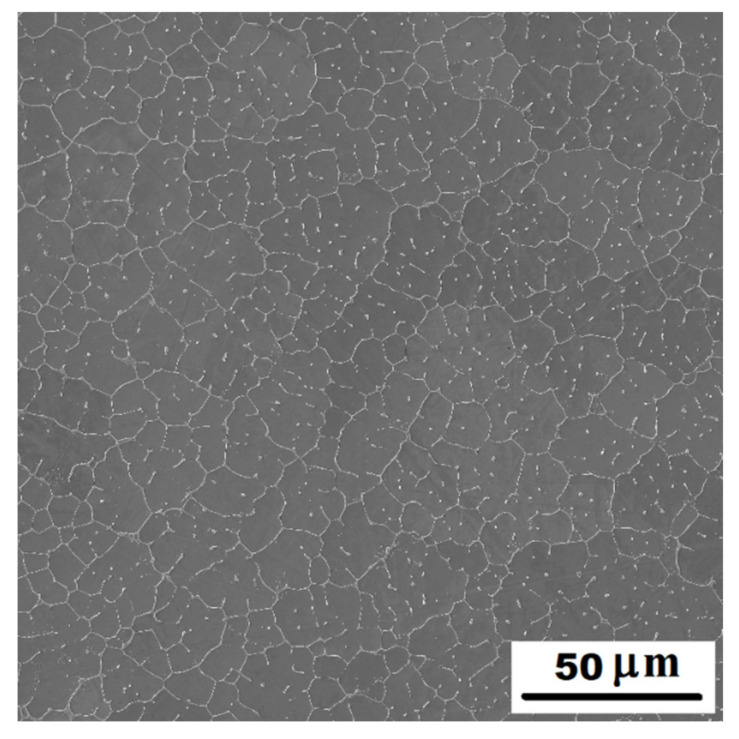
The structure of Fe-28Al-15Si-2Mo alloy in the as-cast state (overview)—SE, 10 kV.

**Figure 2:**
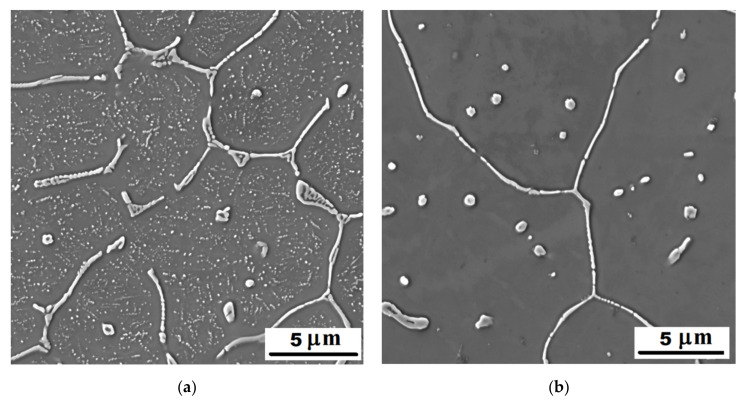
The detail of the structure of Fe-28Al-15Si-2Mo alloy in the as-cast state—SE, 10 kV: (**a**) the place, where the precipitation of very fine particles started during casting; (**b**) the place without fine secondary particles inside the grains.

**Figure 3:**
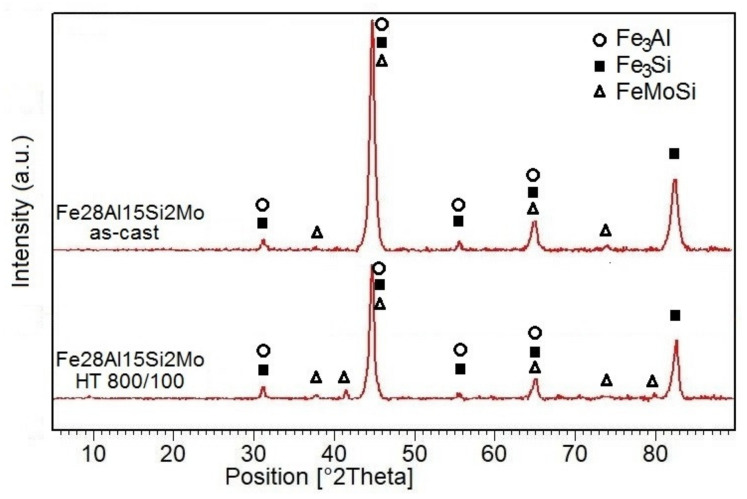
The XRD determination of phases in alloy Fe-28Al-15Si2-Mo (as-cast state and heat-treated state).

In the structure of Fe-28Al-15Si-2Mo as-cast alloy, there were a large number of precipitated secondary particles due to excess of alloying elements (molybdenum and silicon)—see Figure 1 and Figure 2. XRD identified two types of precipitates as Fe–Mo–Si and Fe_3_Si (Figure 3). Based on the comparison of XRD to EDX measurement, Fe–Mo–Si precipitates were present preferably on the cell boundaries. Their shape was needle-like or eutectic-like in some places—see coarser particles in Figure 2a. Coarse globular particles of Fe–Mo–Si were also observed inside the grains—see Figure 2a,b. In places, very fine “chain-arranged” precipitates (Fe_3_Si and also probably Fe–Mo–Si particles, both forming during the casting) were observed—see fine particles inside the grains in Figure 2a. The total volume fraction of all precipitates was calculated at about 5% in areas without fine chain-arranged precipitates inside the grains (see Figure 2b), while in areas with these fine precipitates was calculated at about 15% (see Figure 2a).

**Figure 4:**
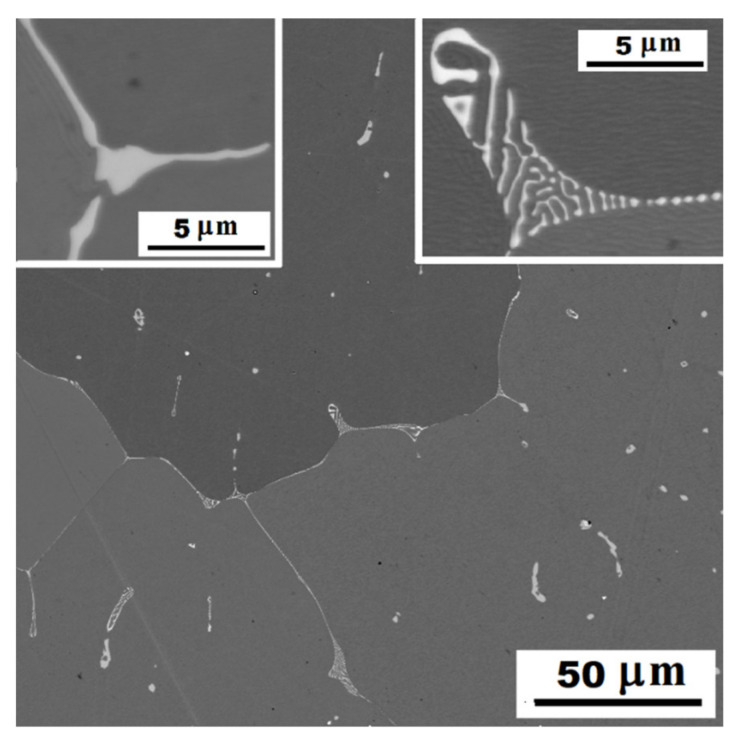
The structure of Fe-28Al-15Si alloy in the as-cast state—SE, 10 kV. Detail left: individual needle-like particles. Detail right: eutectic-like areas on the cell boundaries.

For comparison, the structure of alloy without molybdenum addition (Fe-28Al-15Si as-cast) is stated in Figure 4. The amount of precipitates is significantly lower in the structure of as-cast alloy without molybdenum addition. They are arranged in the form of small eutectic-like areas on the cell boundaries or individual needle-like particles (see details in Figure 4). Comparing Figure 1 and Figure 4, it is clear to see that just a molybdenum addition is responsible for creating large quantities of the secondary phase particles.

**Figure 5:**
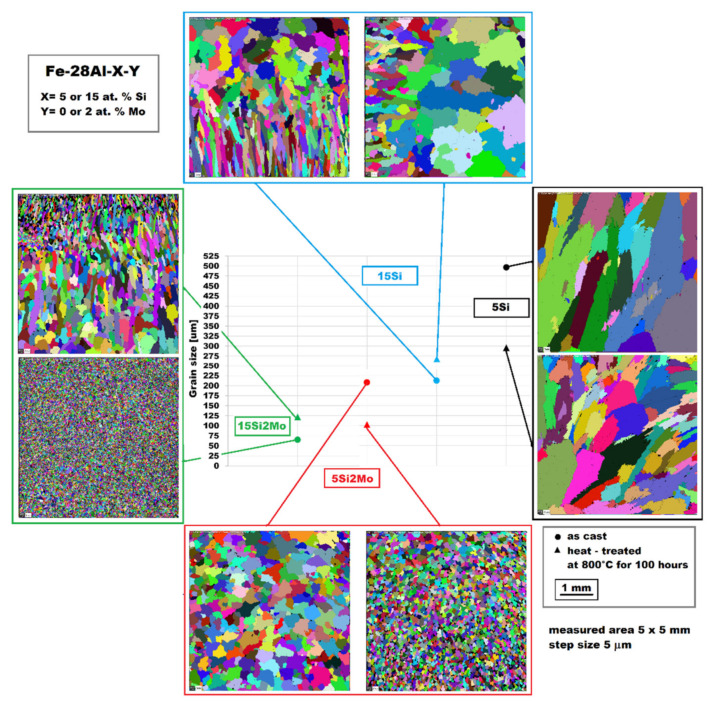
The grain sizes of Fe-28Al-X-Y alloys (X = 5 or 15 at. % Si; Y = 0 or 2 at. % Mo) in as-cast and also in annealed states.

Additionally, it is obvious, from the comparison of structures of alloys with/without molybdenum (taken by EBSD Figure 5), that molybdenum presence is a crucial factor to obtain a fine-grained structure. In Fe-28Al-15Si-2Mo as-cast alloy, there are very fine grains with homogeneous dimensional distribution—65 ± 9 µm, while in the alloy without molybdenum addition (Fe-28Al-15Si as-cast) there are significantly coarser grains—app. 210 µm (see Figure 5). Furthermore, the dimensional distribution of grains is considerably inhomogeneous.

For the alloys with lower silicon content [1], the molybdenum effect is similar—the grains in Fe-28Al-5Si as-cast alloy are coarser, with an average dimension of about 497 µm, while the average grain size is about 208 µm in the structure of Fe-28Al-5Si-2Mo as-cast alloy (Figure 5).

#### 3.1.2. Structure of Alloys in the Annealed State

After annealing at 800 °C for 100 h, the grain coarsening was observed in the case of Fe-28Al-15Si-2Mo alloy—see EBSD Figure 5.

The dimensions of individual grains were not homogeneous compared with as-cast alloy—the average grain size was 122 ± 75 µm. Similarly, the grain coarsening after annealing was also observed in the case of Fe-28Al-15Si alloy (average grain size was 270 µm)—see Figure 5.

**Figure 6:**
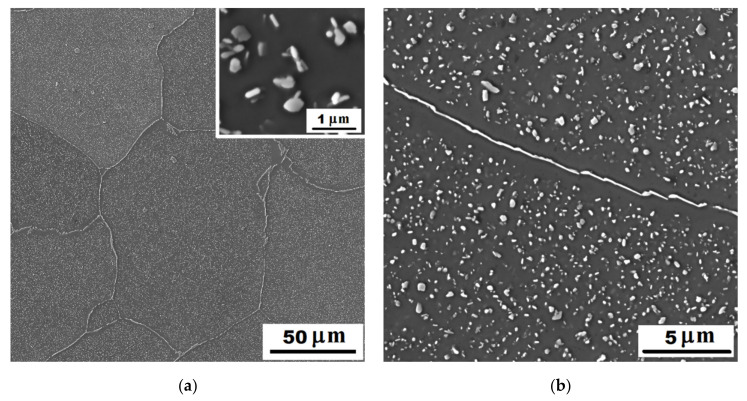
The structure of Fe-28Al-15Si-2Mo alloy after annealing at 800 °C for 100 h—SE, 10 kV: (**a**) overview and detail of very fine precipitates inside the grains; (**b**) the detail of cell boundary.

The structure of Fe-28Al-15Si-2Mo alloy annealed at 800 °C for 100 h is shown in Figure 6a,b. The particles on cell boundaries remain without significant changes in dimensions, shape, or composition (Fe–Mo–Si—XRD Figure 3) in comparison to the as-cast state (see Figure 6a and detail in Figure 6b). The individual globular particles present in the as-cast state inside the grains (Figure 2b) were dissolved into the matrix. On the other hand, the dispersion of nano or sub-micro scale Fe3Si and Fe–Mo–Si precipitates (XRD Figure 3) starts to form very densely inside the grains, after supplying activation energy by heat-treatment (see detail in Figure 6a). The dimensions of these incoherent secondary particles are in the range between 50 and 500 nm. The total volume fraction of all precipitates was calculated at about 15%. Very fine, densely, and homogeneously distributed precipitates can contribute to the matrix significantly reinforcing. However, long needle-like brittle particles on the cell boundaries in connection with the narrow precipitates-free band (see Figure 6b) could reduce the strengthening effect.

**Figure 7:**
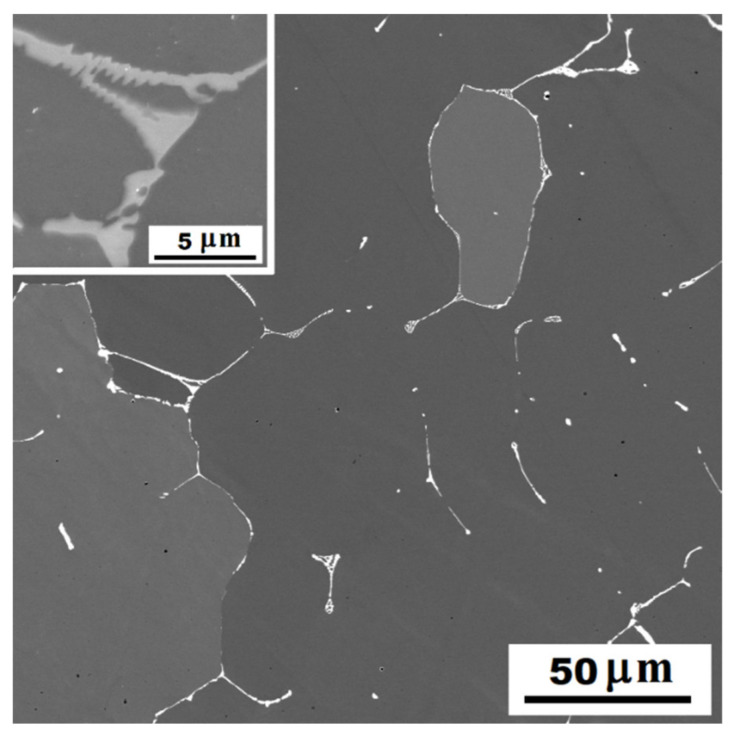
The structure of Fe-28Al-15Si alloy after annealing at 800 °C for 100 h—SE, 10 kV.

The alloy structure without molybdenum addition (Fe-28Al-15Si) shows no significant changes after performed heat treatment (Figure 7).

### 3.2. The High-Temperature Properties

#### 3.2.1. The High-Temperature Yield Stress in Compression

**Figure 8:**
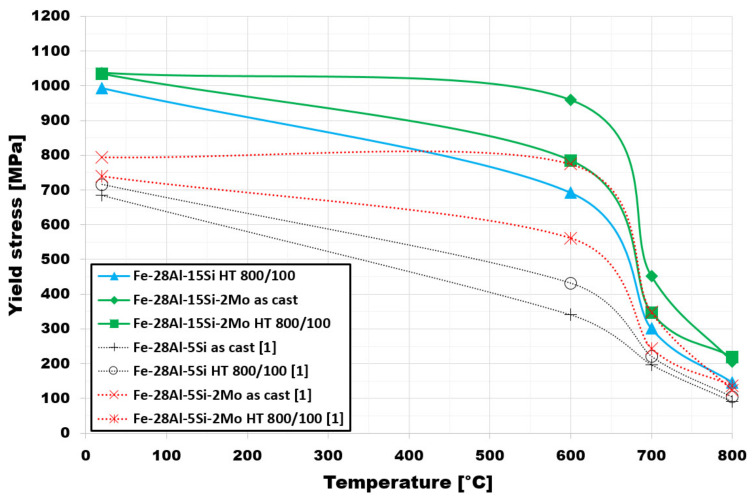
The values of yield stress in compression for investigated alloys as well as for compared alloys [1].

Regarding the high-temperature compression test (Figure 8), the investigated Fe-28Al-15Si-2Mo alloy was compared to alloys with lower silicon content (5 at. %) and with/without molybdenum investigated recently [1].

It is evident that the higher content of silicon, in alloys doped with molybdenum, leads to a significant increasing in values of high-temperature yield stress in compression for as-cast states, mainly at temperatures up to 600 °C. Specifically, the value of yield stress is higher by more than 240 MPa at room temperature and more than 180 MPa at 600 °C in comparison to lower-silicon containing Fe-28Al-5Si-2Mo alloy [1].

The same conclusion is valid in the case of annealed states of these alloys, where the increase represents about 290 MPa at room temperature and 220 MPa at 600 °C in comparison to Fe-28Al-5Si-2Mo alloy [1]. Even at a temperature of 800 °C, the increase in the corresponding value is about 1.6 times.

In comparison to the alloy without molybdenum addition (blue line in Figure 8—Fe-28Al-15Si), it is evident that molybdenum addition also plays a role in the increase of high-temperature yield stress values in compression—the measured values of yield stress in compression are higher for all tested temperatures. The increase is about 100 MPa for 600 °C. The ternary alloy was tested in the annealed state only, mainly due to difficulties connected with the poor machinability of material in the as-cast state.

#### 3.2.2. The Dilatation Behaviour of Fe-28Al-15Si-2Mo and Fe-28Al-5Si-X Alloys

Iron aluminides are materials designed to work at high temperatures, where thermal expansion must be considered. Therefore, detailed knowledge of the coefficient of thermal expansion (CTE) is necessary to determine the dimensional tolerances of machine parts correctly.

For the evaluation of the silicon influence amount, the CTE of the Fe-28Al-15Si-2Mo alloy as well as the CTE of Fe-28Al-5Si-2Mo alloy were measured. For comparison, the influence of the addition of different alloying elements on CTE was shown for Fe-28Al-5Si-2X alloys (X = Ti, Nb, or Mo).

**Figure 9:**
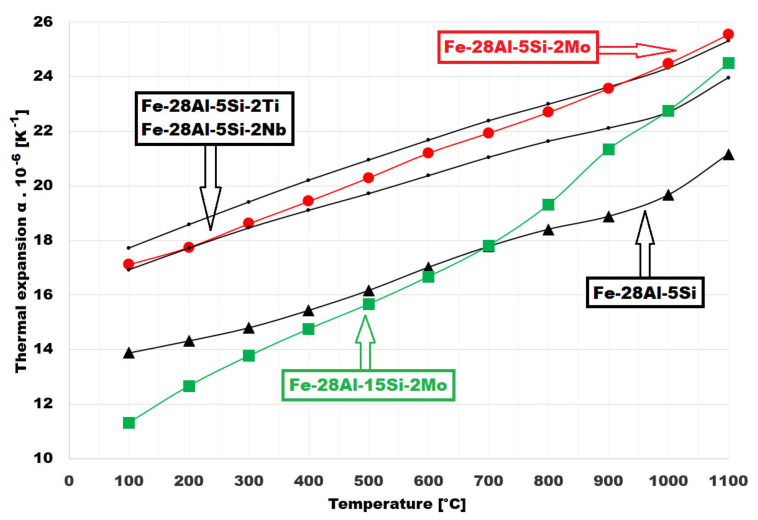
The thermal expansion of Fe-28Al-15Si-2Mo and Fe-28Al-5Si-2X alloys.

The CTE values of all alloys are summarized in Figure 9. It is obvious from the chart that the quaternary “X” element(s) added to the ternary Fe-28Al-5Si alloy has minimal influence on the slope of the thermal expansion curve, but the CTE is higher at about 3 × 10^−6^ K^−1^ in the whole temperature range. The slope of the curve of Fe-28Al-15Si-2Mo alloy is different. This alloy has relatively low CTE at low temperatures (about 11.5 × 10^−6^ K^−1^ at 100 °C). At 1100 °C, the CTE of Fe-28Al-15Si-2Mo is similar to the CTE values of Fe-28Al-5Si-2X (X = Ti, Nb, or Mo).

## 4. Discussion

Two mechanisms appear to be contributing to matrix strengthening in Fe-28Al-15Si-2Mo alloy: the strengthening by solid solution and strengthening by grain boundaries due to very fine grains. The solubility of silicon, as well as molybdenum, is relatively high in the Fe-28Al type matrix. Therefore, a strong reinforcing effect by solid solution hardening can be expected. The contribution of strengthening by solid solution is supposed to be higher compared to alloy with 5 at. % of silicon because of a three-times amount of silicon solute in the matrix. In the as-cast state, the contribution of grain boundaries strengthening can be higher compared to annealed state due to finer grains.

After annealing, the matrix is silicon depleted because silicon is involved in the formation of the secondary phase. The fine dispersion can be formed by both types of secondary phase particles because the secondary phase particle formation can be influenced by molybdenum in two possible ways: (a) molybdenum participates in phase formation directly, so the phase Fe–Mo–Si forms; (b) molybdenum only supports the formation of Fe_3_Si phase, analogically to Si-supported formation of Al_4_C_3_ phase in the case of Fe–Al-based iron aluminides [23]. In case (b), a possible mechanism may be such that molybdenum substitutes iron in Fe_3_Al solid solution so surplus iron can form the Fe_3_Si phase with silicon. Moreover, the grains get coarse after annealing, so the contribution of grain boundaries strengthening is decreasing. The contribution of the secondary phase to strengthening is then not enough to compensate for the impact of the above. From the reasons mentioned, measured values of high-temperature yield stress in compression are lower in the annealed state.

However, the formation of the fine dispersion of the secondary phase during annealing leads to the improvement of the processing properties, mainly machinability. The presence of the fine brittle particles causes a so-called “short chip”, which is favourable for machinability [29].

A further benefit can be considered to be an increase of compressive ductility in the annealed state—this increased from 9.9% in the as-cast state to 15.7% in the annealed state, whereas the yield stress in compression has been the same for both states at room temperature.

It is obvious from the comparison of alloys with and without molybdenum addition (Figure 8), that molybdenum presence also contributes to the increase of yield stress. This fact corresponds with the formation of secondary particles supported by molybdenum alloying in both states (compare Figure 1 and Figure 4 or Figure 6a and Figure 7).

Regarding CTE values, the Fe-28Al-15Si-2Mo alloy has lower CTE at temperatures up to 1000 °C in comparison to alloy with lower silicon content (Fe-28Al-5Si-2Mo)—see Figure 9. In the case of Fe-28Al-15Si-2Mo alloy, there are changes in the direction of the approximation line evident at about 600 and 900 °C. The calculated Equations (1)–(3) of approximation straight lines:

y = 0.0105x + 10.47 for temperature range 100–600 °C (slope of curve 0.0105)(1)

y = 0.0132x + 8.70 for temperature range app. 600–900 °C (slope of curve 0.0132)(2)

y = 0.017x + 5.87 for temperature range 900–1100 °C (slope of curve 0.017)(3)

These curve slope changes correspond with the changes of crystallographic ordering to long-range distance (from D0_3_ to B2 lattice and then from B2 to αFe). For the binary alloy with the corresponding composition (meaning Fe-28Al), the phase transition from D0_3_ to B2 lattice is recorded at 550 °C approximately, and the phase transition from B2 to αFe is noticeable at 900 °C (according to binary Fe—Al diagram). Used alloying elements can shift these transition temperatures. The slower increase of the CTE values during heating is evident at lower temperatures up to 600 °C. This trend can be connected with the properties of the D0_3_ matrix. The volume fraction of precipitates can also affect the CTE values because alloys with a high volume fraction of secondary phase(s) are working as composite materials, in which each phase can have different expansion characteristics.

## 5. Conclusions

Silicon addition in the higher amount (15 at. %) causes the significant increase of values of the high-temperature yield stress in compression of Fe-28Al-15Si-2Mo alloy in the as-cast state compared to alloys with 5 at. % of silicon [1], primarily due to a higher level of solid strengthening solution. Moreover, the presence of molybdenum also contributes to this increase by significant grain refinement.

The annealing of Fe-28Al-15Si-2Mo alloy at 800 °C for 100 h leads to the depletion of matrix by silicon and to the formation of a very fine dispersion of Si-rich precipitates inside the grains. Concurrently, the annealing causes the grain coarsening, so the values of the high-temperature yield stress in compression are lower at 600 °C and 700 °C in comparison to the as-cast state. However, this type of stabilization annealing improves significantly the workability/machinability of alloy doped with 15 at. % of silicon.

The measured coefficient values of thermal expansion are lower for Fe-28Al-15Si-2Mo alloy in comparison to the alloys with 5 at. % of silicon in the temperature range up to 600 °C, considered as the maximal application temperature of this type of alloy.

## Figures and Tables

**Table 1:** The nominal chemical composition of investigated (or compared [1]) alloys.

Alloy	The Nominal Chemical Composition [at. %]			
	Fe	Al	Si	Mo
Fe-28Al-15Si	Bal.	28.0	15.0	−
Fe-28Al-15Si-2Mo	Bal.	28.0	15.0	2.0
Fe-28Al-5Si [1]	Bal.	28.0	5.0	−
Fe-28Al-5Si-2Mo [1]	Bal.	28.0	5.0	2.0

## Data Availability

All data are available from the authors.
